# *Staphylococcus aureus* bacteremia and cardiac implantable electronic devices in a county hospital setting: a population-based retrospective cohort study

**DOI:** 10.48101/ujms.v126.5653

**Published:** 2021-03-05

**Authors:** Sara Pichtchoulin, Ingrid Selmeryd, Elisabeth Freyhult, Pär Hedberg, Jonas Selmeryd

**Affiliations:** a Department of Clinical Physiology, Västmanland County Hospital, Västerås, Sweden; b Department of Infectious Diseases, Västmanland County Hospital, Västerås, Sweden; c Department of Microbiology, Västmanland County Hospital, Västerås, Sweden; d Centre for Clinical Research, Uppsala University, Västmanland County Hospital, Västerås, Sweden

**Keywords:** *Staphylococcus aureus*, cardiac implantable electronic device, endocarditis, pacemaker

## Abstract

**Background:**

Due to a high incidence of cardiac implantable electronic device-associated infective endocarditis (CIED-IE) in cases of *Staphylococcus aureus* bacteremia (SAB) and high mortality with conservative management, guidelines advocate device removal in all subjects with SAB. We aimed to investigate the clinical course of SAB in patients with a CIED (SAB+CIED) in a Swedish county hospital setting and relate it to guideline recommendations.

**Methods:**

All CIED carriers with SAB, excluding clinical pocket infections, in the County of Västmanland during 2010–2017 were reviewed retrospectively.

**Results:**

There were 61 cases of SAB+CIED during the study period, and CIED-IE was diagnosed in 13/61 (21%) cases. In-hospital death occurred in 19/61 (31%) cases, 34/61 (56%) cases were discharged with CIED device retained, and 8/61 (13%) cases were discharged after device removal. Subjects dying during hospitalization were elderly and diseased. No events was seen if the CIED was removed. Among four discharged cases with conservatively managed CIED-IE one relapse occured. Among 30 cases discharged with retained CIED and no evidence of IE, 22/30 (73%) cases had an uneventful follow-up, whereas adverse events secondary to overlooked CIED-IE were likely in 1/30 (3%) cases and could not be definitely excluded in additionally 4/30 (13%) cases.

**Conclusions:**

During the study period, management became more active and prognosis improved. The heterogeneity within the population of SAB+CIED suggests that a management strategy based on an individual risk/benefit analysis could be an alternative to mandatory device removal.

## Introduction

Many elderly in the western world are equipped with cardiac implantable electronic devices (CIEDs), probably >2–3% among subjects aged >75 years, and the number of implanted devices is increasing (1). The incidence of CIED-associated infective endocarditis (CIED-IE) among CIED carriers with *Staphylococcus aureus* bacteremia (SAB) is high and has been estimated to be 27–45% in different studies (2–5). Device removal is strongly recommended for patients with CIED-IE (6–9). Diagnosis of CIED-IE in SAB cases is challenging, and multiple clinical parameters must be weighed together to reach one (9, 10). Also, multiple comorbidities, old age, and short life expectancy complicate the management of such patients (11). Because of the high incidence of CIED-IE in patients with SAB, diagnostic difficulties, and poor prognosis with conservative management, Swedish national guidelines from 2016 have advocated device removal in all subjects with SAB, including cases where CIED-IE cannot be demonstrated (8). The 2020 European Heart Rhythm Association (EHRA) and the 2017 Heart Rhythm Society (HRS) consensus papers have similar recommendations (6, 7). With an evident pocket infection, the diagnostic challenges are minor and management is well established (6). In contrast, the management of SAB in CIED carriers (SAB+CIED), when CIED-IE is not evident upon presentation, is more complex. The objective of this study was to describe the background characteristics, management, and outcome of SAB+CIED, excluding pocket infections, within the County of Västmanland, Sweden, and relate them to current guidelines.

## Materials and methods

All SAB+CIED in the County of Västmanland, Sweden (population of 275,000), from 2010 to 2017 were reviewed retrospectively. Cases of SAB+CIED were identified by cross-linking all hospital-acquired blood cultures positive for *S. aureus* in the database of the microbiology laboratory during 2010–2017 with 1) the Swedish Pacemaker Registry (12) containing data on all pacemakers implanted since 1989 and onward; and 2) the local hospital’s registries of diagnoses according to the International Statistical Classification of Diseases and Related Health Problems (ICD-10) for CIED-related diagnostic codes (T8xx, Z9xx, Z4xx, FPxxx, TFP00, DF016, and DF031) in 2008–2017. For all matches, the presence of a CIED at the time of SAB was verified in the patient’s medical journal. Cases with clinical pocket infections were excluded. Data were collected by reviewing digital hospital medical records from all departments in the County. Background data were collected on age, gender, comorbidities according to the Charlson Comorbidity Score (CCS) (13), residential care status (home dwelling or nursing home residency), general medical conditions according to the highest National Early Warning Score version 2 (NEWS2) (14) during the week following the taking of blood culture, SAB community acquisition (15), type of device (pacemaker, implantable cardioverter-defibrillator [ICD], or cardiac resynchronization therapy [CRT]), and time of implantation and last revision. Information was also collected on diagnostic procedures and treatments: if the diagnosis of CIED-IE was considered, diagnostic imaging, antibiotic treatment, clinical diagnosis, and whether device removal was performed. Each case was classified by the modified Duke criteria (10) based on available clinical information, autopsy results, and lead cultures, into CIED-IE or not. CIED-IE was defined as either lead endocarditis or valvular IE in a CIED carrier. Analogously to previous studies, we defined CIED-IE as the fulfillment of the criteria for *definite* IE (using the criteria for possible IE would have classified all cases as CIED-IE since SAB, as a major criterion, and presence of an intracardiac device, as a minor criterion, are sufficient for possible IE) (3, 4). Similar to previous studies (2–4), the outcome was measured as relapse of SAB or death from any cause during hospitalization or within 90 days of discharge. The rationale for using this timeframe is that SAB episodes occurring >70 days after initial blood culture are much more likely to be reinfection than a relapse (16, 17). The Regional Ethics Board of Uppsala, Sweden, approved the study protocol 2018-11-29 (Dnr 2017/513/1).

### Statistical analyses

Continuous variables are reported as medians and interquartile ranges (IQRs), and categorical variables are reported as counts and percentages. To account for clustering when comparing groups (a few subjects had more than one SAB episode during the study period), confidence intervals (CIs) calculated by generalized linear models with the cluster bootstrap were calculated and compared (18). The clustering effect was small, with negligible differences in results compared to those obtained by more conventional methods. Therefore, approximate independence between observations was assumed, and differences between groups were finally evaluated with the Wilcoxon rank-sum for continuous variables and Fisher’s exact test for categorical variables. To illustrate trends of dichotomous variables graphically over time, predictions based on univariate logistic regression models were plotted with 95% CIs. *P*-values < 0.05 were considered statistically significant. R version 3.6.2 was used for all analyses (https://www.r-project.org). The R packages eulerr and ClusterBootstrap were used in the analysis.

## Results

From the microbiology database, 1,035 unique patients were identified with SAB during 2010–2017. When cross-linked against the National Pacemaker Registry, there were 72 unique patients with 98 SAB episodes. Of these, 66 episodes occurred in subjects where a CIED was in place when afflicted by SAB. After excluding four episodes with clinical pacemaker pocket infections and one episode where the subject was transferred to another hospital early after admission, the study sample consisted of 61 SAB cases in 55 unique patients. We also cross-linked subjects with SAB with pacemaker-related ICD-10 codes from the hospital registry to validate our search strategy. This gave an identical subset of patients except for one, who was present in the National Pacemaker Registry but had no pacemaker-related ICD-10 code registered.

**Figure 1:**
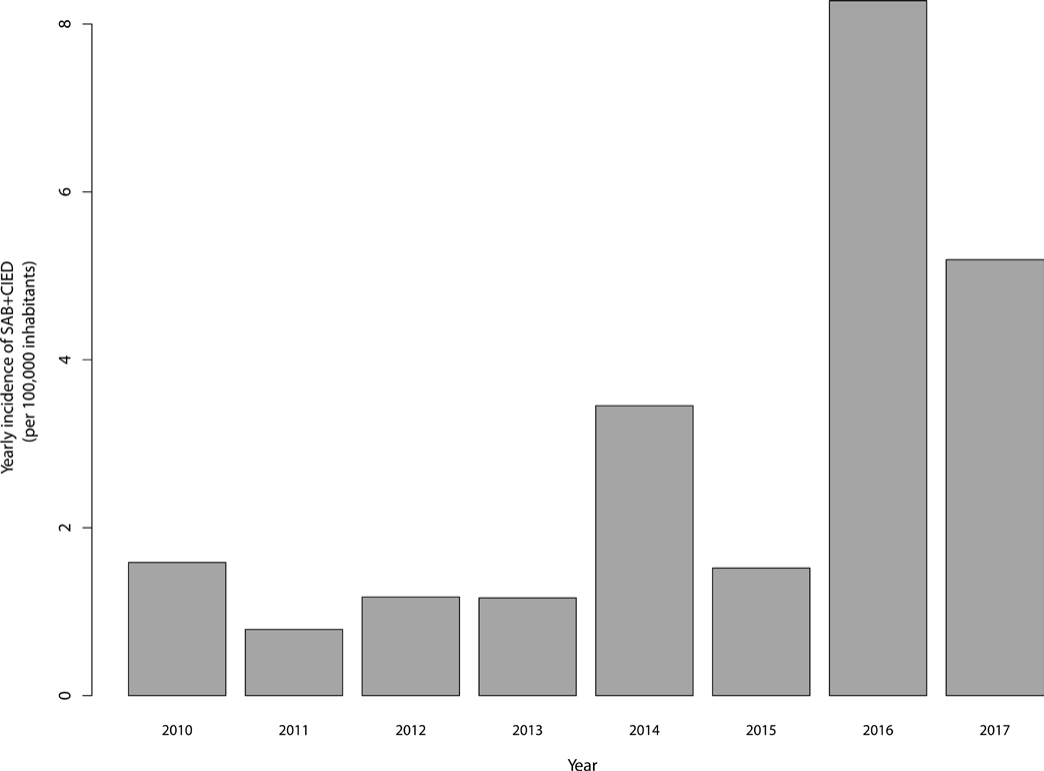
The yearly incidence of hospitalization due to *Staphylococcus aureus* bacteremia in cardiac implantable electronic device carriers (SAB+CIED) in the County of Västmanland, Sweden.

**Figure 2:**
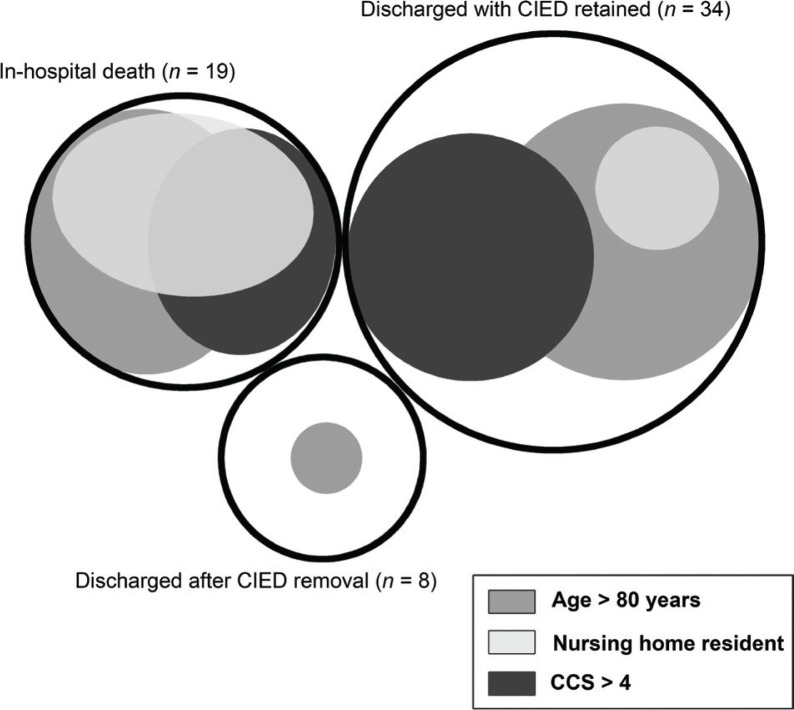
Euler diagrams illustrating the distribution and overlapping of some background characteristics stratified by discharge and device removal status. Medians were used for dichotomization. The sizes of the outer black circles are proportional to the sizes of the subgroups. CIED: cardiac implantable electronic device; CCS: Charlson Comorbidity Score.

**Figure 3:**
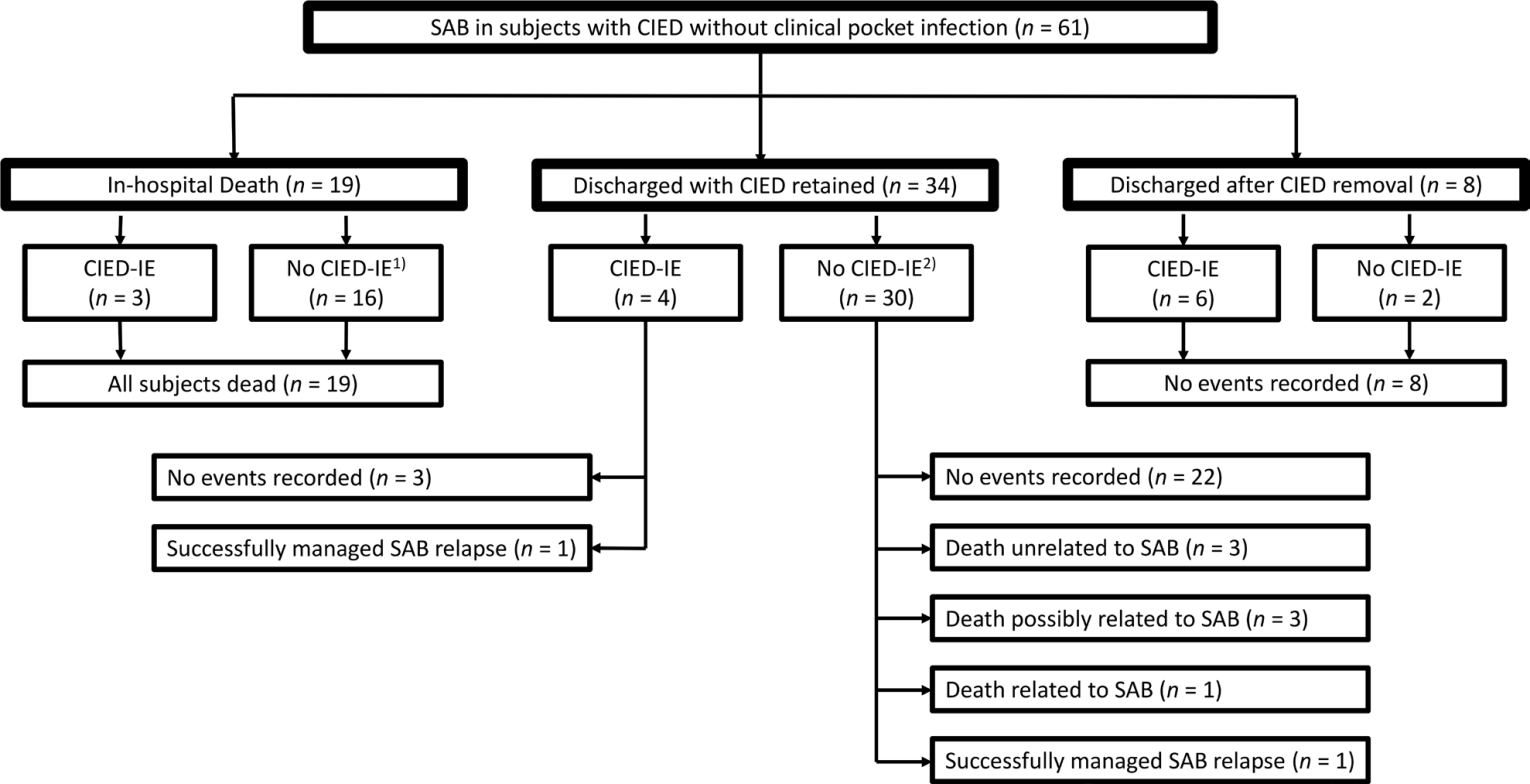
Outcomes stratified by discharge and device removal status. CIED: cardiac implantable electronic device; IE: infective endocarditis; SAB: *Staphylococcus aureus* bacteremia. 1) Echocardiography was not performed in 13 cases. 2) Echocardiography was not performed in six cases.

The incidence of SAB+CIED increased with time (Figure 1). Basic characteristics, outcome, and management stratified by discharge and CIED removal status are illustrated in Tables 1 and 2, Figures 2 and 3, and Supplementary Figure 1. In the study sample, 37/61 (61%) cases were male, and the median age was 80 years (IQR 73–85). One case had methicillin-resistant *S. aureus* (MRSA), whereas the majority of cases had methicillin-susceptible *S. aureus* (MSSA). A clinical diagnosis of CIED-IE was established in 13/61 (21%) cases. One of these cases did not fulfill the Duke definite criteria but was clinically diagnosed as CIED-IE, despite negative transthoracic echocardiography (TTE), in the context of recurring SAB after a recent CIED-IE diagnosis.

**Table 1:** Characteristics of cases with SAB+CIED (*Staphylococcus aureus* bacteremia+cardiac implantable electronic device) stratified by in-hospital death and discharge status.

Variable	All cases (*n* = 61)	In-hospital death (*n* = 19)	Discharged (*n* = 42)	*P*^a^
Male	37 (61)	15 (79)	22 (52)	0.088
Age	80 (73–85)	86 (79–90)	77 (73–83)	<0.001
Age > 80 years	29 (48)	13 (68)	16 (38)	0.051
Charlson Comorbidity Score (CCS)	4 (3–5)	5 (3–5.5)	4 (2–5)	0.153
CCS > 4	22 (36)	10 (53)	12 (29)	0.089
National Early Warning Score 2 (NEWS2)	7 (6–10)	10 (7.5–13)	6 (5–8)	<0.001
Symtomatic days before blood culture (BC)	1 (0–3)	2 (1–4.5)	1 (0–2.8)	0.148
Nursing home resident	13 (21)	10 (53)	3 (7.1)	<0.001
Methicillin-resistant *S. aureus* (MRSA)	1 (1.6)	0 (0)	1 (2.4)	1.000
Type of CIED				0.703
Cardiac resynchronization therapy (CRT) or CRT-D	7 (11)	1 (5.3)	6 (14)	
Implantable cardioverter-defibrillator (ICD)	6 (9.8)	2 (11)	4 (9.5)	
Pacemaker	48 (79)	16 (84)	32 (76)	
Acquisition				0.394
Community aquired	14 (23)	3 (16)	11 (26)	
Healthcare associated	30 (49)	12 (63)	18 (43)	
Nosocomial	17 (28)	4 (21)	13 (31)	
Known non-infective endocarditis (IE) focus	22 (36)	4 (21)	18 (43)	0.151
Implantation (years)	6.8 (3.8–11)	7.2 (3.9–13)	6.7 (3.9–9.6)	0.503
Last revision or implantation (years)	4.6 (2.7–7.8)	4.6 (2.7–7.6)	4.6 (2.8–7.5)	0.950
Possibility of CIED-IE discussed	26 (43)	6 (32)	20 (48)	0.276
Transthoracic echocardiography (TTE) or transesophageal echocardiography (TEE) performed	42 (69)	6 (32)	36 (86)	<0.001
TEE performed	10 (16)	0 (0)	10 (24)	0.023
CIED-IE diagnosis	13 (21)	3 (16)	10 (24)	0.737

Values are counts and (percentages) for categorical variables and medians (with interquartile range) for continuous variables.

a Wilcoxon rank-sum test or Fisher’s exact test.

**Table 2:** Characteristics of SAB+CIED (*Staphylococcus aureus* bacteremia+cardiac implantable electronic device) cases discharged from hospital stratified by CIED removal status.

Variable	All discharged cases (*n* = 42)	CIED removed (*n* = 8)	CIED retained (*n* = 34)	*P*^a^
Male	22 (52)	5 (62)	17 (50)	0.700
Age	77 (73–83)	73 (66–74)	79 (73–83)	0.061
Age > 80 years	16 (38)	1 (12)	15 (44)	0.127
Charlson Comorbidity Score (CCS)	4 (2–5)	1.5 (1–2.2)	4 (3–5.8)	0.002
CCS > 4	12 (29)	0 (0)	12 (35)	0.080
National Early Warning Score 2 (NEWS2)	6 (5–8)	6.5 (4.8–8.2)	6 (5–7.8)	0.783
Symtomatic days before blood culture (BC)	1 (0–2.8)	2.5 (0–4.5)	1 (0–2)	0.379
Nursing home resident	3 (7.1)	0 (0)	3 (8.8)	1.000
Methicillin-resistant *S. aureus* (MRSA)	1 (2.4)	1 (12)	0 (0)	0.190
Type of CIED				0.031
Cardiac resynchronization therapy (CRT) or CRT-D	6 (14)	1 (12)	5 (15)	
Implantable cardioverter-defibrillator (ICD)	4 (9.5)	3 (38)	1 (2.9)	
Pacemaker	32 (76)	4 (50)	28 (82)	
Acquisition				0.005
Community aquired	11 (26)	5 (62)	6 (18)	
Healthcare associated	18 (43)	0 (0)	18 (53)	
Nosocomial	13 (31)	3 (38)	10 (29)	
Known non-infective endocarditis (IE) focus	18 (43)	1 (12)	17 (50)	0.109
Implantation (years)	6.7 (3.9–9.6)	4.3 (2–8.1)	7 (4.3–11)	0.210
Last revision or implantation (years)	4.6 (2.8–7.5)	3.7 (2–5.1)	5 (3.3–7.5)	0.222
Possibility of CIED-IE discussed	20 (48)	8 (100)	12 (35)	0.001
Transthoracic echocardiography (TTE) or transesophageal echocardiography (TEE) performed	36 (86)	8 (100)	28 (82)	0.576
TEE performed	10 (24)	4 (50)	6 (18)	0.075
CIED-IE diagnosis	10 (24)	6 (75)	4 (12)	0.001
Outcome				0.473
No event	33 (79)	8 (100)	25 (74)	
Death from any cause	6 (14)	0 (0)	6 (18)	
Relapse	3 (7.1)	0 (0)	3 (8.8)	

Values are counts and (percentages) for categorical variables and medians (with interquartile range) for continuous variables.

a Wilcoxon rank-sum test or Fisher’s exact test.

### Death during hospital care

In-hospital death occurred in 19/61 (31%) of cases. These cases were older (86 vs. 77 years; *P* < 0.001), were more frequently nursing home residents (53% vs. 7%; *P* < 0.001), and were in a worse general condition, as reflected by a higher NEWS2 score (10 vs. 6; *P* < 0.001), compared with cases discharged from the hospital. Of the cases dying during hospital care, 8/19 (42%) died within 3 days of care, TTE was performed in 6/19 (32%) cases, and transesophageal electrocardiography (TEE) was performed in none. Three cases were diagnosed with CIED-IE with vegetations on a native valve, a prosthetic valve, and a CIED lead, respectively. None of these cases were in a clinical state where endocarditis surgery or CIED extraction was deemed feasible. Only two of the cases without CIED-IE diagnosis were autopsied (without evidence of CIED-IE).

### Discharged after CIED removal

Of the 42/61 (69%) cases discharged from hospital, the CIED was explanted in 8/42 (19%) cases. These cases were younger (73 vs. 79 years; *P =* 0.061) and had lower CCS (1.5 vs. 4; *P =* 0.002) compared with those discharged with a retained CIED. There were no relapses or deaths in the explanted cases. In 5/8 cases, clinical Duke definite criteria were fulfilled and lead cultures were positive in two and negative in three cases, but this was after >4 days of intravenous (i.v.) antibiotics. In 3/8 cases, clinical Duke definite criteria were not fulfilled: one subject with fever 3 h postimplantation of an ICD device (lead culture not taken); one 58-year-old patient with severe heart failure, CRT, and persistent bacteremia on day 3 (negative lead culture after >4 days of i.v. cloxacillin); and finally, one 63-year-old patient with persistent bacteremia on day 3 and a recently implanted pacemaker where an indication for pacemaker treatment no longer existed (positive lead culture).

### Discharged with CIED retained

Among the 34/61 cases (56%) discharged with a retained CIED, 4/34 (12%) had a diagnosis of CIED-IE. These conservatively treated CIED-IE patients received 6 weeks of i.v. and/or peroral (p.o.) antibiotic suppression treatment. There were no deaths among cases of conservatively treated CIED-IE. However, one relapse was detected in an 80-year-old woman with lymphoma where a CIED lead infection had been proven, but device removal was deemed unfeasible. She received 45 days of i.v. antibiotics and relapsed 42 days postdischarge, after which she was put on chronic suppressive antibiotics.

Among the 30 cases without a CIED-IE diagnosis, there were two SAB relapses: there was one early relapse in an 86-year-old man with chronic obstructive pulmonary disease (COPD) and heart failure, 9 days after the termination of a 12-day treatment with i.v. cloxacillin in the context of a negative TTE. This patient did not survive his relapsing SAB. Another patient, an 83-year-old woman with metastatic cancer under palliation, had 14 days of i.v. cloxacillin and a negative TTE and relapsed 80 days postdischarge. The relapse was treated with a short course of i.v. antibiotics, and the patient lived another 4 months without apparent signs of infection. There were six deaths without microbiologically documented SAB relapse. In three cases, blood cultures were negative for *S. aureus* upon clinical deterioration, and plausible alternative death causes were documented: gastrointestinal bleeding, Gram-negative sepsis, and heart failure secondary to terminal cardiac amyloidosis. In three cases, blood cultures were not taken, and undiagnosed SAB relapse could not be ruled out: one 92-year-old female patient on chronic suppressive p.o. antibiotics for an *S. aureus* hip prosthesis infection died in her dementia nursing home 8 days after discharge; one 87-year-old male patient with COPD died 10 days after discharge in respiratory and cardiac failure without signs of infection; one 72-year-old male patient with dilated cardiomyopathy died 27 days after discharge in his home.

### Trends during the inclusion period

**Figure 4:**
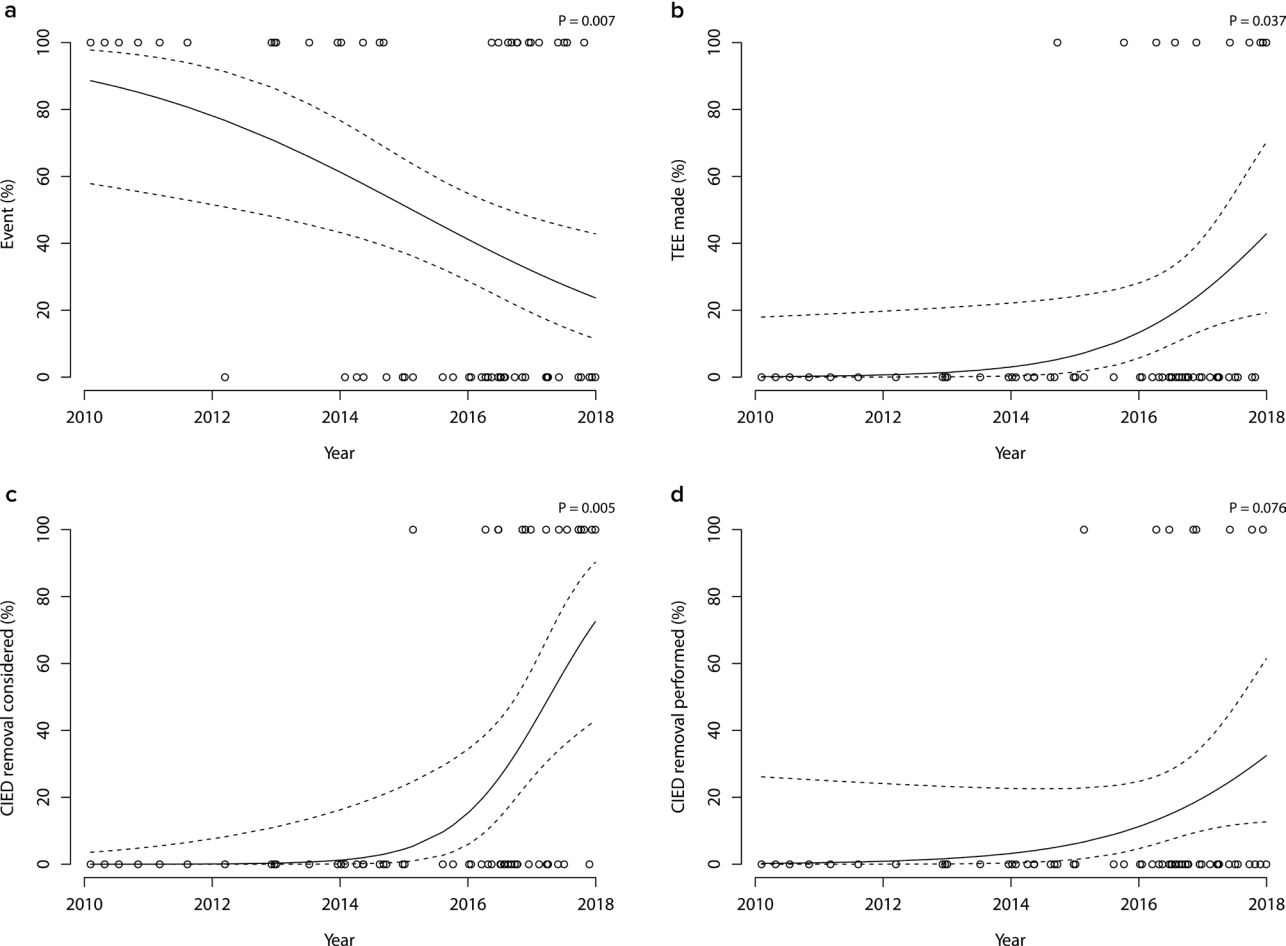
Trends in outcome and management during the study period. The probabilities of having an adverse event (a), having transesophageal echocardiography (TEE) performed (b), cardiac implantable electronic device (CIED) removal considered (c), and CIED removal performed (d), expressed as functions of the year of inclusion are illustrated by unadjusted logistic regression probability estimates. Individual measurements are indicated as circles. *P*-values were calculated by logistic regression for association with the year of inclusion.

As illustrated in Figure 4 and Supplementary Figures 2 and 3, management, basic characteristics, and outcomes were not constant over the inclusion period 2010–2017. When comparing the period 2010–2013 with 2014–2017, the risk of having an adverse event decreased from 92 to 35% (*P* < 0.001) and in-hospital mortality from 67 to 22 % (*P =* 0.005). There was no significant association between inclusion time and age, CCS, NEWS2 score, or nursing home residency. With regard to management, device removals were more frequently considered, 48–96 h blood cultures were more frequent, and there were trends toward referring for more echocardiography and device removals.

## Discussion

Here, we have described the background characteristics, management, and outcome of all cases of SAB+CIED without signs of pocket infections between 2010 and 2017 in a county hospital. The incidence of hospitalizations due to SAB+CIED increased steeply during the study period.

In the most recent 2016 national Swedish guidelines on IE management, the indication for device removal was broadened by recommending it for all subjects with SAB, regardless of diagnostic findings (8). The profound effect these recommendations had on management can be seen in Figure 4C, where device removal from 2016 and later, was considered in a majority of patients. However, the recommendations seem to have been challenging to implement in real life as only a fraction of patients were finally explanted.

Among the 34 cases discharged with a retained CIED, 30 had no CIED-IE diagnosis. Most of these (22/30; 73%) had an uneventful follow-up, but among the six deaths and two relapses, there might have been cases of missed CIED-IE. Based on available clinical information, an overlooked CIED-IE was probable in one case with early relapse, possible in four cases (late relapse or early death with very limited clinical data), and unlikely in three (negative blood cultures and/or no clinical signs of infection at the time of death). If all cases of nonproven CIED-IE had been explanted, in line with current guideline recommendations, 25–29 out of 30 (83–97%) device removals would, retrospectively, have been carried out in vain, whereas it would potentially have been advantageous in 1–5 out of 30 cases (3–17%). This potential benefit needs to be compared with the potential harm associated with device removal. Lead extraction is a safe procedure in general, but for specific subgroups, the risk of major complications and death is increased to >5% (19–21). The risk of adverse outcome increases, for instance, with active sepsis, multiple leads, female gender, long lead dwell time, implantation at young age, multiple previous CIED procedures, and anemia (19–21). As the potential benefits and risks might be of similar gross magnitude for specific subgroups, a strategy based on an individual risk/benefit analysis might be preferable over mandatory device removal: if the probability of CIED-IE is high (i.e. persistent bacteremia or positive imaging) and the risk of complications is low (i.e. low procedural risk), CIED removal would be warranted, whereas a low CIED-IE probability and/or a high risk of complications might favor a more conservative approach. Several risk scoring systems have been put forward that potentially could be used to identify subjects with an elevated risk of adverse outcome with extraction (19–21). Unfortunately, scoring systems for predicting CIED-IE in SAB+CIED are scarce. To our knowledge, only one exists, the PREDICT-SAB, which, however, does not take imaging findings, or lack thereof, into account (4). With PREDICT-SAB, subjects without any risk factors, including persistent bacteremia, had a predicted probability for having CIED-IE of 7% before imaging. Similarly, in a recent study, subjects with negative imaging findings and without persistent bacteremia were shown to have a relapse rate of 5% with CIED retention, and it was suggested that CIED retention might be a viable option in such subjects if the risk with extraction was increased (22).

The CIED-IE prevalence in the present study was 21% compared with 27–34% in previous studies performed in tertiary referral centers (device pocket infections excluded) (2–4). A referral bias, where subjects with a high CIED-IE probability are transferred and concentrated to tertiary referral centers, could in part explain this. However, another likely explanation was probably underdiagnosis because of the infrequent utilization of diagnostic imaging. For instance, the subjects dying during hospital care were rarely evaluated for the possibility of CIED-IE, as illustrated by a TTE frequency of 33% and TEE frequency of 0%. While this might be perceived as negligent, it is likely the result of rapid clinical deterioration in the patients (42% of them died within 3 days) and/or a short expected survival not perceived to be modifiable by diagnostic or therapeutic approaches because of advanced age (median age 86 years), frailty (53% were nursing home residents), multiple comorbidities (median CCS 5), and a poor general condition (median NEWS2 score 10). Also, many of the cases were managed prior to contemporary awareness of CIED-IE.

In this study, 46% of the cases had an adverse event (relapse or death from any cause within 90 days). The overall mortality was 41%, in-hospital mortality was 31%, and relapse rate was 5%. These values are similar to the outcomes in previous studies on similar populations; for example, Uslan et al. reported in-hospital mortality of 32% (3), Chamis et al. reported an all-cause mortality of 36% and a relapse rate of 6% (2), and Sohail et al. described an all-cause mortality of approximately 40% (4). Nevertheless, the similarities in mortality and relapse rates with the current study are surprising given that our population was considerably older (80 years vs. 70–73) (2–4), there was a lower utilization of diagnostic modalities (TEE 16% vs. 64–67%) (2–4) and a lower frequency of device removal (13% vs. 36%) (2). One reason for this pattern might be a bias toward the referral of complicated cases with a high suspicion of CIED-IE to tertiary referral centers. Also, MRSA was very uncommon in the present population compared with the abovementioned studies (2% vs. 39–55%) (2–4), which might explain a better than expected prognosis in our population as MSSA is associated with better outcomes (23). Recently, in a similarly designed Swedish study on SAB in 33 CIED carriers, Snygg-Martin et al. demonstrated frequencies of device removal (12%), echocardiography (63%), TEE (33%), and mortality (30-day: 36%; 1-year: 65%) very similar to those of the present study (5).

During the study, the prognosis for patients improved substantially (Figure 4a), consistent with previous findings for SAB (24), *S. aureus* IE (25), and sepsis cohorts (26–28). There are probably several explanations for this. We observed signs of more active management strategies over time, as exemplified by more use of echocardiography and more device removals. The large impact national IE guidelines published in 2016 (8) had on management can be appreciated in an increased awareness of CIED-IE and utilization of 48–96 h blood cultures (Supplemental Figure 3d). However, the onset of the improvement in prognosis seemed to precede the shifts in management, indicating other factors at play. Changes in population characteristics might have contributed, even though the relative importance of such factors seems to be less pronounced as no significant linear association between age, NEWS2 score, CCS, nursing home residential status, community acquisition, lead dwelling time and type of CIED with time could be detected (Supplementary Figures 2 and 3). Improvements in different SAB care processes, not analyzed specifically here – for instance, increased sepsis awareness, standardization of management (29), a shortened time to antistaphylococcal therapy (24), and improvements in intensive care management (28) were likely to be essential factors in explaining the observed improvements seen in prognosis.

## Limitations

There were several important limitations to our study. First, the restricted sample size and number of events made it impossible to analyze the effect of multiple background factors and management on the outcomes while adjusting for confounding effects. Second, as observations were cases and not subjects, the assumption of independence among observations was violated to a degree. However, the violation was deemed to be of minor importance as only a few within-subject repeated observations were present, and analysis accounting for clustering yielded similar results (data not shown). Third, the long inclusion time of 8 years “diluted” the results over the years, which hampered our ability to focus on and describe current practices accurately. Fourth, any generalizability of results must be done with caution because of the study’s single-center county hospital setting. Fifth, since most cases were included before the contemporary CIED-IE criteria of the European Society of Cardiology (ESC) and EHRA, we chose to use the older modified Duke criteria frequently employed in previous SAB+CIED studies. The main difference between ESC/EHRA criteria and the modified Duke criteria is additional diagnostic modalities (intra-cardiac ultrasound, leukocyte scintigrahy, gated computer tomography, and positron emission tomography) within the imaging major criteria. However, as these novel imaging modalities were not implemented in clinical practice during the study period and as the weighting of criteria has not changed, the classification would have been similar to the ESC/EHRA criteria. Sixth, the reported prevalence of CIED-IE in this study was likely underestimated because of the infrequent utilization of diagnostic imaging.

## Conclusions

During the study period, management of SAB+CIED became more active, paralleling new guideline recommendations, and the patients’ prognosis improved, illustrating the importance of the active management advocated by current guidelines. However, the heterogeneity of the population concerning age, comorbidities, and outcomes and a low frequency of SAB relapses among subjects discharged with CIED retained suggest that, instead of device removal in all SAB+CIED, a strategy based on an individual risk/benefit analysis of device retention vis-à-vis removal might be an option. However, further research on the development and validation of clinical prediction tools to aid in such risk/benefit analysis is needed.
